# Allele-Level *KIR* Genotyping of More Than a Million Samples: Workflow, Algorithm, and Observations

**DOI:** 10.3389/fimmu.2018.02843

**Published:** 2018-12-04

**Authors:** Ines Wagner, Daniel Schefzyk, Jens Pruschke, Gerhard Schöfl, Bianca Schöne, Nicole Gruber, Kathrin Lang, Jan Hofmann, Christine Gnahm, Bianca Heyn, Wesley M. Marin, Ravi Dandekar, Jill A. Hollenbach, Johannes Schetelig, Julia Pingel, Paul J. Norman, Jürgen Sauter, Alexander H. Schmidt, Vinzenz Lange

**Affiliations:** ^1^DKMS Life Science Lab, Dresden, Germany; ^2^DKMS, Tübingen, Germany; ^3^San Francisco School of Medicine, University of California, San Francisco, San Francisco, CA, United States; ^4^Department of Internal Medicine I, University Hospital Carl Gustav Carus at the Technische Universität Dresden, Dresden, Germany; ^5^Division of Biomedical Informatics and Personalized Medicine, and Department of Immunology, University of Colorado Anschutz Medical, Aurora, CO, United States

**Keywords:** next generation sequencing, NGS, *KIR* genotyping, *KIR*, killer cell immunoglobulin-like receptors, allele-level resolution, high-throughput

## Abstract

The killer-cell immunoglobulin-like receptor (*KIR*) genes regulate natural killer cell activity, influencing predisposition to immune mediated disease, and affecting hematopoietic stem cell transplantation (HSCT) outcome. Owing to the complexity of the *KIR* locus, with extensive gene copy number variation (CNV) and allelic diversity, high-resolution characterization of *KIR* has so far been applied only to relatively small cohorts. Here, we present a comprehensive high-throughput *KIR* genotyping approach based on next generation sequencing. Through PCR amplification of specific exons, our approach delivers both copy numbers of the individual genes and allelic information for every *KIR* gene. Ten-fold replicate analysis of a set of 190 samples revealed a precision of 99.9%. Genotyping of an independent set of 360 samples resulted in an accuracy of more than 99% taking into account consistent copy number prediction. We applied the workflow to genotype 1.8 million stem cell donor registry samples. We report on the observed *KIR* allele diversity and relative abundance of alleles based on a subset of more than 300,000 samples. Furthermore, we identified more than 2,000 previously unreported *KIR* variants repeatedly in independent samples, underscoring the large diversity of the *KIR* region that awaits discovery. This cost-efficient high-resolution *KIR* genotyping approach is now applied to samples of volunteers registering as potential donors for HSCT. This will facilitate the utilization of *KIR* as additional selection criterion to improve unrelated donor stem cell transplantation outcome. In addition, the approach may serve studies requiring high-resolution *KIR* genotyping, like population genetics and disease association studies.

## Introduction

The human killer cell immunoglobulin-like receptor (*KIR*) gene family resides on the long arm of human chromosome 19 in a region that varies between 100 and 200 kb in size. This region is characterized by highly diverse haplotypes, that differ both in gene content and copy number of the *KIR* genes (1–5). *KIR* genes encode transmembrane glycoproteins, expressed on the surface of natural killer (NK) cells (6, 7), which are an important component of the innate immune system and provide a first line of defense against infectious agents and tumor cells (8). KIR are key modulators of NK cell activity with activating and inhibiting family members. Activating KIR induce target cell killing upon receptor stimulation, and inhibitory KIR counteract NK cell activation thereby playing an important part in the detection of HLA downregulation: a common mechanism of immune evasion of virally infected cells or tumor cells (9, 10).

The importance of the interactions between KIR and HLA molecules has been well established: *KIR* and *HLA* class I have been found to be actively co-evolving (11, 12). KIR binding specificity and affinity to HLA class I molecules, and consequently patterns of resistance to specific diseases are influenced by complex interactions of allelic polymorphisms of both *KIR* and *HLA* class I genes (13–18).

Successful allogeneic hematopoietic stem cell transplantation (HSCT) crucially depends on a close match of *HLA* class I and class II alleles between donor and recipient (19), and *HLA* matching remains the most important criterion for unrelated donor selection (20). Yet, consideration of additional factors such as *KIR* may further improve transplantation outcome. Several studies reported an influence of donor *KIR* genotype on long-term survival after transplantation (21–27). Owing to the fact that *KIR* and *HLA* genes reside on different chromosomes, unrelated donors and recipients who are *HLA*-matched rarely share identical *KIR* genes. Genotyping the *KIR* genes of potential stem cell donors at high resolution upon registration and providing this information in addition to the *HLA* genotypes would thus be beneficial for optimizing the unrelated donor selection process (28, 29).

Due to the complexity of the human *KIR* region, high-resolution genotyping is not straightforward. There are 15 *KIR* genes and two *KIR* pseudogenes named (30). In this study we will refer to this classical *KIR* gene nomenclature, even though *KIR3DL1*/*KIR3DS1* and likewise *KIR2DL2*/*KIR2DL3* constitute alleles of the same gene (31). On the other hand, we treat *KIR2DL5A* and *KIR2DL5B*, which are highly homologous genes allocated to clearly distinct genomic locations (32), as a single gene. Six *KIR* genes (*KIR2DL1, KIR2DL2*/*3, KIR2DL5, KIR3DL1, KIR3DL2, KIR3DL3*) encode inhibitory glycoproteins with long (L) cytoplasmic tails, while six genes encode glycoproteins with short (S) cytoplasmic tails that induce activation (*KIR2DS1, KIR2DS2, KIR2DS3, KIR2DS4, KIR2DS5, KIR3DS1*). *KIR2DL4* exhibits both activating and inhibitory functions (33). Two pseudogenes are not expressed as proteins (*KIR2DP1, KIR3DP1*). Four genes are present in most of the common haplotypes and have been designated “framework” genes (*KIR3DL3, KIR3DP1, KIR2DL4, KIR3DL2*). They mark the centromeric and telomeric boundaries of the *KIR* region (2, 11). The *KIR* region shows extensive genetic diversity at three distinct levels (2, 5, 11). First, *KIR* haplotypes vary with respect to gene content, i.e., the presence or absence of specific *KIR* genes on a haplotype (2, 11). Next, some *KIR* genes are subject to copy number variation (CNV), having up to four copies on a single haplotype (5). Finally, individual *KIR* genes show extensive sequence polymorphism with currently 907 alleles being described in total (IPD-KIR Database, Release 2.7.1) (30). Allelic diversity across genes ranges from 16 alleles for *KIR2DS3* and *KIR2DS1* to 158 alleles for *KIR3DL2*.

Established *KIR* genotyping methods focus on assessing *KIR* gene content, i.e., patterns of presence or absence for individual *KIR* genes (34, 35). Methods that report CNV or allelic differences have been developed (5, 31, 36), but they are mostly designed to serve the needs of the research community and do not support the cost and scale requirements for high-volume stem cell donor registry typing.

DKMS registers volunteers for stem cell donation across Germany, Poland, the USA, the UK, and Chile. Since 2013, the high-throughput genotyping facility of DKMS (DKMS Life Science Lab, Dresden, Germany) has been applying next generation sequencing (NGS) for high-throughput *HLA* genotyping (37, 38). After initially developing this NGS-based workflow for six *HLA* genes we extended the donor typing profile by adding *CCR5* and the blood groups *ABO* and *RHD* (38–40). Here, we describe the workflow and the algorithm used to provide cost-effective high-volume *KIR* genotyping at allelic resolution. We compare the results to established *KIR* genotyping methods and present an analysis of *KIR* allele frequencies based on a subset of more than 300,000 samples.

## Materials and Methods

### Aim

Our short-amplicon-based NGS workflow for *HLA* genotyping achieves extreme cost efficiency for high-throughput genotyping by omitting the classical NGS library preparation steps. Instead, short amplicons are designed to match the instrument sequencing length. Upon pooling all targeted loci, molecular indexes and adapters required for sequencing are added using a second PCR reaction. Here, we describe the workflow and the algorithm used to provide *KIR* genotypes at allelic resolution. The output gives full gene presence/absence, copy number and allele data represented in the GL string format (41), which is a standard that enables communication of results among clinics and researchers (42).

### Samples and Consent to Participate

Volunteers from Germany, Poland, USA, and UK provided samples to DKMS for registration as potential HSCT donors. *HLA* and *KIR* genotyping was performed on these samples between October 2016 and August 2018. All subjects gave written informed consent for *HLA* and *KIR* genotyping in accordance with the Declaration of Helsinki. The described genotyping is within the scope of the consent forms signed at recruitment and performed as a genotyping service. UCSF sample collection with written informed consent (accuracy assessment based on 360 samples) was approved by the Institutional Review Board of the University.

### DNA Isolation and Quantification

DNA was isolated from 150 μl whole blood or a single nylon “FLOQSwab ™ hDNA free” (Copan Italia Spa, Brescia, Italy) using the magnetic-bead-based “chemagic DNA Blood Kit special” or “chemagic DNA Buccal Swab kit special” (Perkin Elmer, Baesweiler, Germany), respectively. DNA was eluted in 100 μl elution buffer (10 mM Tris-HCl pH8.0). DNA concentrations were measured by fluorescence (SYBR Green, Biozym, Hessisch Oldendorf, Germany) using the TECAN infinite 200 Pro (Tecan, Männedorf, Switzerland) plate reader. Samples with DNA concentrations of <2 ng/μl were excluded from *KIR* genotyping.

### Reference Samples With *KIR* Genotyping Information

We obtained genomic DNA from a panel of 93 International Histocompatibility Working Group (IHWG) cell lines (43). The samples were diluted to a concentration of 20 ng/μl. An additional set of 360 reference samples were derived from a collection of healthy individuals stored in the UCSF multiple sclerosis biorepository. Both sample sets had been previously genotyped for *KIR* at allelic resolution (31).

### Amplicon Design and PCR Amplification

Primers were designed to target *KIR* exons 3, 4, 5, 7, 8, and 9. As exon 8 is very short (53 bp) and close to exon 9 a combined amplicon was designed for these two exons. Primer mixes contained 10–14 primers per amplicon to cover all *KIR* genes in a multiplexed assay. Amplicon sizes varied between 249 and 448 bp. Four separate PCR reactions (exon 4, exon 5, exons 3, and 7 multiplexed, one amplicon spanning exons 8 and 9) were performed in 10 μl volume using 384-well plates with FastStart™ Taq DNA Polymerase (Roche, Basel, Switzerland) and the associated buffer system (38). For each sample, the four PCR reactions were pooled using volumes appropriate to obtain balanced read coverage for each *KIR* exon. A secondary PCR was performed on the pool to elongate the amplicons with indexes and sequencing adapters for Illumina sequencing as described before (38). Target-specific primers and index primers were obtained from Metabion (metabion international AG, Planegg, Germany).

### Library Preparation and Sequencing

After the indexing PCR, 384 barcoded samples were pooled together. Pooled PCR products were purified with SPRIselect beads (BeckmanCoulter, Brea, USA) with a ratio of 0.6:1 beads to DNA and subsequently quantified by qPCR using the Library Quant Illumina Kit (KAPA Biosystems, Boston, USA). Commonly, 10 purified and quantified *KIR* amplicon pools were combined with 10 amplicon pools targeting the *HLA*, blood group and *CCR5* genes as described previously (38). Denaturation and dilution of the sequencing library were executed as recommended by Illumina (MiSeq Reagent Kit v2-Reagent Preparation Guide). Libraries usually consisting of 3,840 samples were loaded at 12.5 pM onto HiSeq flow cells with 10% PhiX spiked in. Paired-end sequencing was performed for 2 times 249 cycles with HiSeq Rapid SBS Kits v2 (500 Cycles) on HiSeq2500 (Illumina, San Diego, USA) instruments.

### Nextype for *KIR*

The general design of the neXtype software for *KIR* allele-level genotyping follows the design of neXtype for *HLA* genotyping as described previously (37). However, there are two major challenges for short-amplicon-based *KIR* genotyping: First, gene content varies depending on the haplotypes. Second, some *KIR* genes show very high sequence homology either partially or completely with other *KIR* genes. Therefore, particular exonic sequences may not only be shared across alleles of the same *KIR* gene but also across two or more distinct *KIR* genes. Hence, some fundamental assumptions of our established *HLA* genotyping algorithm do not hold true: For *KIR* genotyping we cannot assume that one (homozygous) or two (heterozygous) sequences are identified for each amplified locus. Instead, the number of distinct sequence features per exon varies from sample to sample.

**Figure 1:**
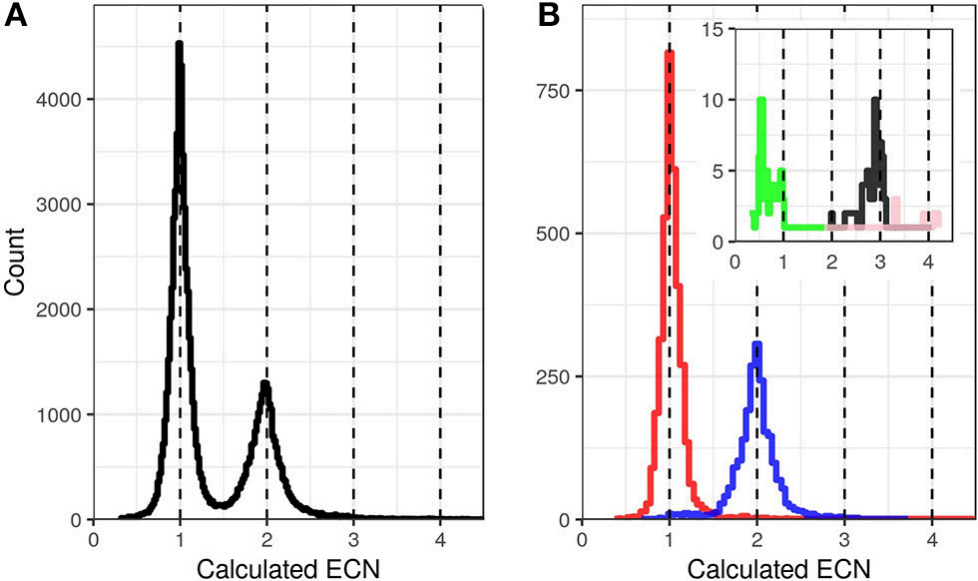
Exon Sequence Copy Number (ECN) calculation based on relative read coverage. Distribution of the calculated ECNs of all exon sequences of 1,000 randomly selected samples **(A)** and 93 reference samples with known KIR genotypes **(B)**. The ECNs were calculated for all detected exon sequences and plotted as events in histograms with a bin size of 0.02. For **(B)** the events were separated and color coded based on the known ECNs according to the reference genotypes with ECN = 0 (green, inlay), ECN = 1 (red), ECN = 2 (blue), ECN = 3 (black, inlay), and ECN = 4 (pink, inlay).

These circumstances increase the algorithmic challenge to distinguish between original sample sequences, and PCR or sequencing artifacts. The most abundant sequencing artifacts are PCR-crossover products and single base errors. In *HLA* genotyping, these can easily be identified by their reduced read coverage compared to at most two original allele sequences. In *KIR* genotyping, however, the natural spread of read coverage and the variation in sequence features present require a different strategy. To deal with this and other challenges (described below), we determine the genomic copy number (ECN, Exon-sequence Copy Number) of individual sequence features by taking advantage of the multiplexed amplification of all *KIR* genes in a single PCR reaction per amplicon. This allows calculating the read count of each individual sequence feature in reference to the read count of other sequences, after correcting for preferential amplification (see below). Applying this normalization algorithm, artifacts are clearly separated from single copy sequences (Figure 1). ECN estimation becomes less accurate with increasing copy numbers (Figure 1). However, only one and two copies per haplotype are common, whereas three and more copies are rather rare (5). The ECN estimation is used for four purposes:

1. **Identification of artifacts**. The ECN estimation outperforms simple absolute or relative thresholds because of the underlying calibration algorithm.
2. **Restriction of the result space**. The ECN estimation decreases the number of valid combinations of exonic sequences, particularly for sequences shared across several *KIR* genes. In these cases the number of theoretically valid exon combinations is often very high. By incorporating the ECN estimations many of these combinations can be eliminated.
3. **Determination of gene copy number**. Based on the ECNs the copy number of *KIR* genes can be determined even if the same allele is present in several copies.
4. **Calculation of a quality score**. For every *KIR* gene a quality score is calculated based on the deviation of the calculated copy numbers from the allocated copy numbers (copy number used) for this solution across all exons. This quality score was identified as the most effective means for the identification of erroneous results in our experiments.

Taken together, the determination of the ECNs and their incorporation into the algorithm reduce the result space and improve accuracy for allele-level *KIR* genotyping.

neXtype analyses the sequencing data in seven consecutive steps:

#### Step1: Amplicon Assignment

Based on the primer sequences each read is assigned to one of the five *KIR* amplicon groups (exon3, exon4, exon5, exon7, exon8/9) as described before (37).

#### Step2: Allele Matching

Using the extant IPD-KIR Database information, the sequences of alleles that are indistinguishable at a given exon are gathered into Exon Allele Groups (EAGs). The allele assignment for this study was based on the IPD-KIR Database, Releases 2.6.1 and 2.7.1 (44). For each exon, these EAG sequences are assembled into a decision tree (37).

The exon-assigned reads are mapped to their corresponding decision trees allowing mismatches. After mapping all reads, the algorithm delivers a set of EAGs and the number of reads mapped to each EAG. We term those EAGs that share their sequence across different genes as “bridging” EAGs. Bridging EAGs cannot be assigned *a priori* to a single gene.

#### Step3: EAG Classification

After all reads are matched, EAGs are classified based on read counts, patterns of sequence mismatches and other statistical properties (Table 1). EAGs identified at this point as artifacts are flagged and eliminated from subsequent analyses.

**Table 1:** Classification of detected sequence features (EAGs).

**Classification**	**Description**	**Used in copy number calculation (“Result EAG”)**
Actual result	Bona fide result not otherwise classified	Yes
Potential new allele	The majority of matched reads deviates from the reference sequence in at least one position	Yes
Statistical noise	Using a binomial distribution an EAG with few associated reads can be identified as noise of an EAG with more reads	No
Potential crossover artifact	The EAG may potentially have been arisen as crossover artifact of two EAGs with more reads	Yes
Crossover artifact	Negligible crossover artifact with low number of matched reads	No
Artifact	EAG was identified as a known artifact	No

#### Step4: Copy Number Calculation

Applying calibration parameters experimentally derived for each batch of PCR primers (see below), the copy number calculation converts the read count for each EAG to an exon sequence copy number (ECN_c_). The gene copy number (GCN_c_) is calculated from the average of the (non-bridging) ECN_c_ over the exons. Note that ECN_c_ and GCN_c_ are rational numbers and used without rounding in the following calculations.

#### Step5: Exon Combination

In this step, *KIR* alleles are called by “exon combination.” In this process gene-specific EAGs are combined and compared to the known IPD-KIR Database allele sequences. Only EAG combinations (EAGCs) corresponding to an allele in the IPD-KIR Database are accepted. EAGCs are further combined into EAGC sets, which represent all the potential combinations of alleles present in the genotype. The number of EAGCs (alleles) in a valid EAGC set is constrained by the rounded calculated gene copy number (GCN). To allow for uncertainty in GCN estimation, EAGC sets based on GCN ± 1 are considered.

The decision on which of the EAGC sets represents the final result for a gene is based on least squares scores. This score incorporates the relative difference between the used (ECN_u_) and calculated (ECN_c_) exon copy numbers per EAGC set. Each set is allocated a score *S* which is defined as

$$S=\frac{1}{\sum_{i=EAG_{1}}^{EAG_{n}}ECN_{c}^{i}}\sum_{i=EAG_{1}}^{EAG_{n}}\frac{{\left(ECN_{u}^{i}-ECN_{c}^{i}\right)}^{2}}{ECN_{u}^{i}}$$

where EAG_1_ to EAG_n_ comprise all member EAGs of an EAGC set and, in addition, EAGs detected but not part of the evaluated EAGC set. Consequently, unused EAGs (ECN_u_ = 0) increase the score because the sum over the squares comprises these EAGs (ECN_u_ in the denominator is set equal to 1 in that case). For normalization, the squared error is divided by the sum of all detected ECN_c_ which equals approximately the GCN multiplied by the number of exons analyzed. The set with the smallest score is identified as the best result. In some cases several results have identical scores. Such ambiguities are reported in the GL string and we refer to them as phasing ambiguities.

**Figure 2:**
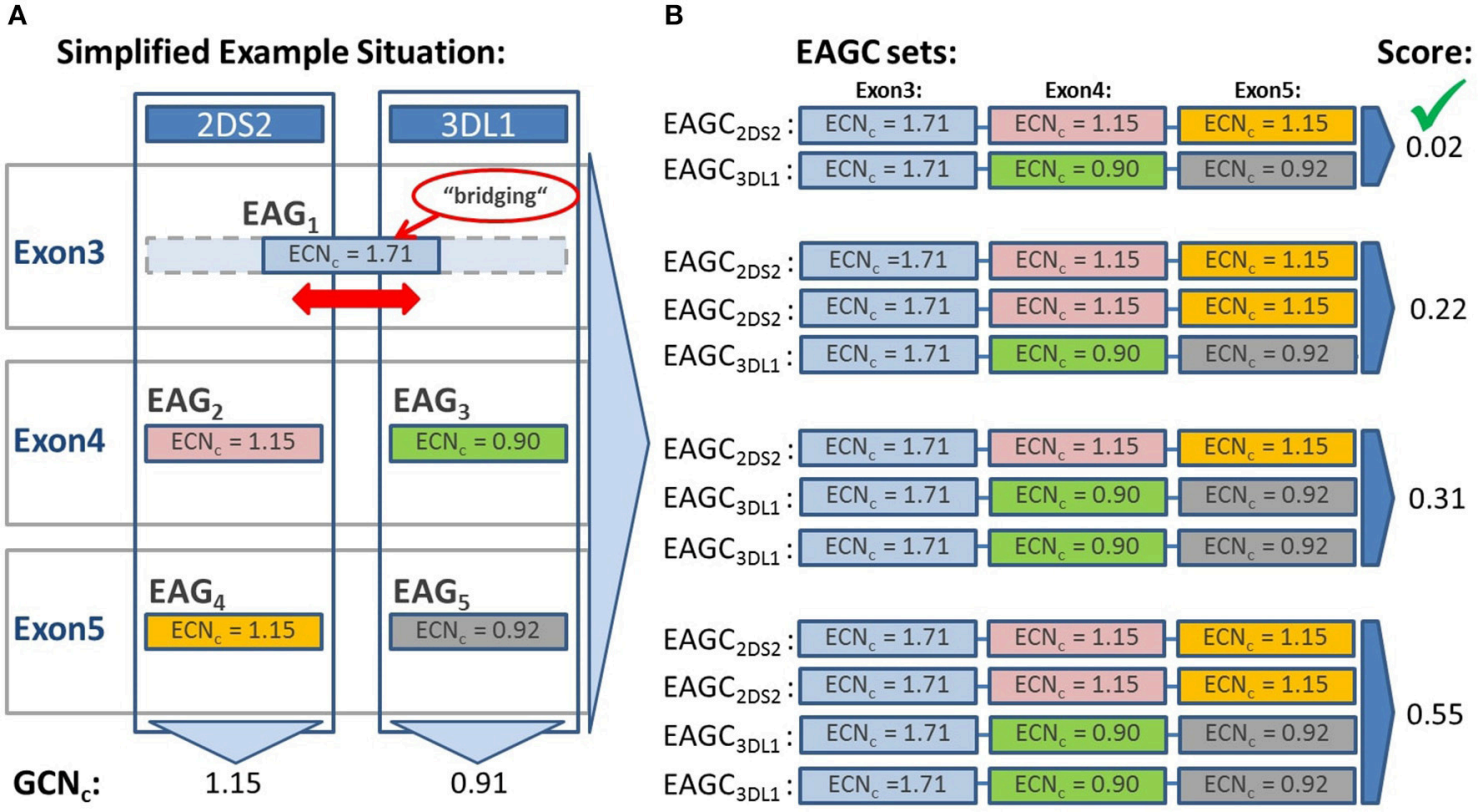
Evaluation of result sets based on the calculation of the result set score S. The result set score S is calculated to evaluate potential results and identify the most likely one. Each result set is composed of different combinations of detected exon sequences (EAGs). This example depicts three exons of two genes sharing in exon 3 the same sequence (bridging EAG). **(A)** EAGs identified in each exon with associated Exon Sequence Copy Numbers (ECNcs). The ECNcs are derived from the number of detected reads by applying several normalization and correction factors. The gene copy number (GCNc) is estimated for each gene by first building the sum of the ECNcs of all EAGs in each exon and then averaging the sums over the exons (ignoring exons with bridging EAGs). **(B)** Based on the detected EAGs neXtype calculates result sets (EAGC sets) consisting of EAG combinations (EAGCs) which correspond to named alleles. In this example EAGC sets with one or two copies per gene are evaluated because the calculated gene copy number (GCNc) is close to 1 for both genes. The EAGC set with one copy of each gene yields the lowest score of 0.02 and is therefore selected as final result.

In the case of bridging EAGs, we consider the respective genes jointly when building EAGC sets. A simplified example is shown in Figure 2. The bridging set with the lowest overall score is chosen as the final result.

#### Step6: Result Rating

The exon combination results are subjected to the following sanity checks:

1. Is a pre-defined minimum read number obtained for each exon?
2. Are all framework genes present?
  Results not passing checks 1 or 2 are blocked and flagged for repeated experimental analysis but may be approved in step 7 after analyst review.
3. Is the copy number of each of the framework genes equal to 2?
4. Are the GCNs of the best results identical?
  Results not passing checks 3 or 4 are flagged for manual verification.
5. Is the score S of each EAGC set < = 0.1?
6. Are all EAGs with an ECN_c_ > 0.6 included in the result set?
7. Is no EAG with ECN_c_ < 0.4 included in the result set?
8. Is ECN_c_-ECN_u_ < 0.4 for the majority of used EAGs?
9. Is no potential new allele included in the result set?

Results not passing checks 5–9 trigger fallback to presence/absence (POS/NEG) calling of the corresponding gene. To report presence/absence of a gene, the GCN_c_ is evaluated: For GCN_c_ > 0.5 the gene is reported as present, otherwise as absent.

The final result is represented in terms of a genotype list (GL) string (41) for each gene, for example:

$$\begin{array}{l} KIR2DL4^{*}00502+KIR2DL4^{*}013|KIR2DL4^{*}0010201/\\ \;KIR2DL4^{*}0010202+KIR2DL4^{*}011. \end{array}$$

This GL string for the gene *KIR2DL4* represents a copy number of two with two possible realizations (genotype ambiguity, denoted by “|”). In one realization one copy has an allele ambiguity (denoted by “/”). To report presence or absence of a gene, “POS” or “NEG” are used in the GL string.

#### Step7: User Interaction

neXtype allows the user to orchestrate the result if required. All classified EAGs not included in one of the EAGC are displayed in addition to the results. The user can add one of those EAGs or flag an EAG as artifact and start a recalculation. It is also possible to exclude a complete exon from the calculation, which may yield a higher level of ambiguities. In addition, the GCNs can be changed manually.

#### Determination of the *KIR* Calibration Parameters

We derive the ECN_c_s from the relative read count of an individual EAG vs. all reads of the exon. However, certain sequences, representing particular genes or alleles, are amplified more efficiently than others. To account for EAG, gene and batch-dependent amplification differences, we introduce three calibration parameters FACTOR_AI, FACTOR_1, and FACTOR_2. For each new reagent batch these parameters are empirically determined by processing at least 1,000 samples (calibration set) including the 93 IHWG samples with known *KIR* genotype.

As described above the reads are assigned to EAGs. As a first calibration step, read counts per EAG are normalized by the total number of reads identified for the respective exons.

To these normalized read counts, FACTOR_AI is applied to compensate for amplification imbalances between EAGs of the same gene and exon. This calibration parameter is derived for each EAG by empirically determining the peak density corresponding to ECN = 1 based on plots of the normalized read counts of the calibration set.

Next, FACTOR_1 is applied to compensate for amplification imbalances between genes and exons. This calibration parameter is derived for each gene and exon (with the calibration set) by applying *k*-means clustering (45) to the read counts, normalized by the previous two steps. FACTOR_1 is then derived from the centroid coordinates of the cluster, empirically determined to correspond to ECN = 1.

Finally, to fine-tune calculated ECNs, FACTOR_2 is applied. To derive FACTOR_2 during parameter determination, ECNs are estimated for each EAG based on reads counts normalized by the previous three steps. These estimates are clustered by applying *k*-means clustering. FACTOR_2 is then derived from the centroid coordinates of the clusters, empirically determined to correspond to ECN = 1 for each locus and exon.

The performance of the calibration parameters is reviewed by manual inspection of density plots derived from the final ECN estimates. Here, the presence of one to two peaks per EAG that correspond to either ECN = 1 or ECN = 2 is verified. Further, the accuracy of typing results of samples with known genotype is another criterion for the approval of a set of calibration parameters for routine typing.

### Characterization of Novel Alleles

To characterize novel alleles for submission to KIR-IPD we applied a redundant full-gene sequencing workflow as previously described for the characterization of novel HLA alleles (46). Briefly, the novel allele was amplified in two independent KIR gene specific long-range PCRs covering the gene from UTR to UTR using PrimeSTAR GXL DNA Polymerase (Takara, Kyoto, Japan). One PCR product was sequenced on Illumina MiSeq instruments after fragmentation and library preparation. The other PCR product was sequenced on PacBio Sequel instruments as unfragmented long amplicon product. The DR2S software (https://github.com/DKMS-LSL/DR2S) was applied to derive fully phased high-quality error-corrected sequences, incorporating both long-read and short-read data (47). The final sequence was submitted to ENA and subsequently KIR-IPD using Typeloader (48).

## Results

### Validation, Precision, Accuracy

Given the complexity of *KIR* genotyping at the allelic level, thorough verification of the workflow and algorithm is essential. A major hurdle is the lack of reference material genotyped to the same depth and breadth. During setup, testing, validation, and optimization of the workflow and algorithm we extensively took advantage of 93 IHWG reference samples that had been previously genotyped for all *KIR* genes at allelic resolution (31). To estimate the precision, we experimentally analyzed a random set of 190 samples 10 times. Finally, we estimated the accuracy of our *KIR* typing approach by analyzing a further set of 360 samples genotyped at *KIR* allele-level.

#### Initial Validation on 93 IHWG Reference Samples

For initial validation of the workflow and algorithm, we analyzed 93 reference samples with predetermined allelic *KIR* genotypes (31). Analysis was performed blinded. Given 16 analyzed *KIR* genes, those 93 samples equal 1,488 genotypes on the level of the individual KIR genes that could feature none, one, or several alleles for each gene. *KIR* genes not present in the respective samples according to the reference data were correctly flagged as absent in 413 cases. In 224 cases (15%) genes were correctly flagged as present, but no allele level was reported (The analysis software reports presence instead of allele-level results if novel alleles are identified or results do not meet all quality criteria). Eight hundred fifty-one genotypes were reported at the allelic level: 848 were in concordance with the pre-typing or have been confirmed after revisiting the reference data. Concordance criteria were: (a) identical alleles called or, in case of not fully resolved alleles, overlap in the allele sets reported in the GL string; (b) identical copy numbers of the alleles called. The three discordant results (0.2%) were due to two cases of *KIR2DL4* with incorrect allele calls and one discrepancy in the copy number of *KIR2DL5*: three copies of an allele were erroneously called instead of two in a homozygous sample. The two *KIR2DL4* cases resulted from a noise filter that disregarded true sequences in rare circumstances, resulting in incorrect allele calls. This filter, inherited from the *HLA* analysis, was found to be not required due to the quantitative approach of the *KIR* algorithm. It was relaxed in the subsequent release to exclude this error source.

#### Assessment of Precision Based on 10-Fold Repeated Analysis of 190 Samples

**Figure 3:**
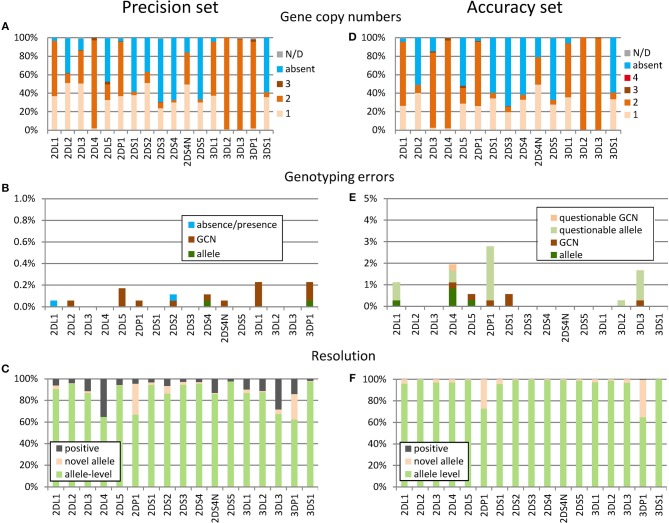
Assessment of precision and accuracy. **(A–C)** Precision set: 10-fold analysis of 190 samples. **(D–F)** Accuracy set: analysis of 360 samples with independent KIR genotyping data. **(A,D)** Gene copy numbers (GCN) based on the established consensus genotypes **(A)** and the corrected results **(D)**. In very few cases the GCN could not be determined unambiguously (gray), partly due to the presence of hybrid genes. **(B,E)** Genotyping errors based on discrepancies between individual replicates and the manually curated consensus genotypes **(B)** or the ground truth reference genotypes **(E)**. Discrepancies are labeled as “questionable” **(E)** if the reference genotypes are not plausible based on manual inspection of neXtype data. **(C,F)** Rate of allele-level results and novel alleles: to avoid spurious results the algorithm reports only the presence of individual KIR genes instead of the exact alleles if certain quality criteria are not met (positive). For the accuracy set **(D–F)**, presence calls were largely improved to allele-level genotypes after manual inspection of the data.

To assess the precision of workflow and algorithm, we randomly selected 190 samples and performed the *KIR* genotyping analysis ten times with this sample cohort, varying instruments, and reagent batches (using KIR-IPD 2.6.1 as reference). Based on the manually curated consensus results we determined the gene copy numbers (Figure 3A). In 6% (12 out of 190) of the samples, at least one of the *KIR* genes was present in three copies, underscoring the need for algorithms able to cope with that complexity. Overall, we obtained results passing quality filters for 1,752 analyses corresponding to 28,032 *KIR* genotypes considering 16 *KIR* genes. (For technical reasons we distinguish in Figure 3 between the expressed and non-expressed variants of *KIR2DS4*, but refer to it jointly as one *KIR2DS4* genotyping result.) Within those 28,032 *KIR* genotypes there were only 19 (0.07%) with discordant results (Figure 3B). Of these, 15 were due to discrepancies in the copy number of the reported alleles, mostly (13/15) discrepancies regarding copy numbers of two or three. In two cases, discrepant alleles were called either instead of the correct allele (*KIR3DP1*) or in addition *(KIR2DS4)*. In two further cases an absent *KIR* gene was falsely called as present (*KIR2DL1* and *KIR2DS2*).

Novel alleles were identified in 5.3% (160 of 3,040) of the consensus genotyping results on the individual gene level (Figure 3C). However, this average is inflated by the two pseudogenes *KIR2DP1* and *KIR3DP1* having several rather frequent alleles not contained in KIR-IPD 2.6.1. Not considering these genes, 2.1% (57 of 2,660) of the genotyping results included novel alleles.

To prevent erroneous calls, neXtype software reports the presence of a gene (positive) instead of distinct alleles if certain quality criteria are not met. The rate of allele level calls was similar for most genes with *KIR2DL4* and *KIR3DL3* being the exceptions (Figure 3C). A particular common artifact in exon 7 was identified as chief cause for *KIR2DL4* underperformance. *KIR3DL3* presence calls were mostly due to a neXtype reference data issue due to missing intron information.

Taken together, we obtained 99.9% concordant *KIR* genotyping results. The data further suggests that the KIR-IPD database currently is far from covering the alleles present in this cohort of 190 samples comprehensively.

#### Assessment of Accuracy Based on the Analysis of 360 Samples

In March 2017 we received an independent set of 360 samples previously analyzed for *KIR* allele-level genotype with the PING software pipeline (31). Validation and precision estimation were set up to mimic high-throughput operations with largely automated analysis. In contrast, these samples were analyzed meticulously as appropriate for clinical samples or clinical research studies. Therefore, samples were processed twice and neXtype results were manually curated. In particular, all neXtype presence calls were reverted to allele-level results with the exception of two cases of *KIR3DP1* (Figure 3F). Data analysis was performed blinded, i.e., without knowledge of PING genotyping data. After data exchange, we worked closely together to define a ground truth based on in-depth inspection of discordant *KIR* typing results.

Overall, copy number distribution in this sample set resembled the 190 sample set data, with 6% of samples harboring three or four copies of specific *KIR* genes (Figure 3D). The frequency of novel alleles was found to be similar to the precision set: Averaging over all *KIR* genes, novel alleles were identified in 5.6% of the results (324 of 5,760). Excluding the two pseudogenes *KIR2DP1* and *KIR3DP1*, novel alleles were identified in 2.0% of the results (101 of 5,040).

The dataset included 360 samples corresponding to 5,760 genotyping results, considering 16 *KIR* genes. However, no reference data was available for *KIR3DP1* (all samples), *KIR2DS2* (all samples), *KIR2DL3* (147 samples) and specific other genes (21 samples) reducing the data set to 4,872 results with PING data. The neXtype genotypes were in concordance with PING genotypes in 99.3% of the cases (Figure 3E). In 11 cases (0.2%) neXtype genotyping errors were identified, including 6 cases of GCN discrepancies and 5 cases of miscalled alleles. In addition, in 21 cases (0.4%) we were not able to establish a ground truth as the two genotyping workflows delivered conflicting results that could not be resolved. In conclusion, this analysis of an independent sample set underlines the potential of the method for high accuracy studies: In this setting it delivered 99.9% allele-level genotypes, including the report of novel alleles, with an accuracy exceeding 99%.

### *KIR* Genotyping of a Large Cohort

Between October 2016 and August 2018 we generated allele-level *KIR* genotyping data for 1.8 million potential HSCT donor samples from Germany (1.08 M), Poland (0.38 M), USA (0.20 M), and UK (0.17 M). Here we report on some characteristic features of a subset of 337,387 samples of this predominantly European cohort analyzed using the most recent KIR-IPD database version (2.7.1) as the reference allele set.

**Figure 4:**
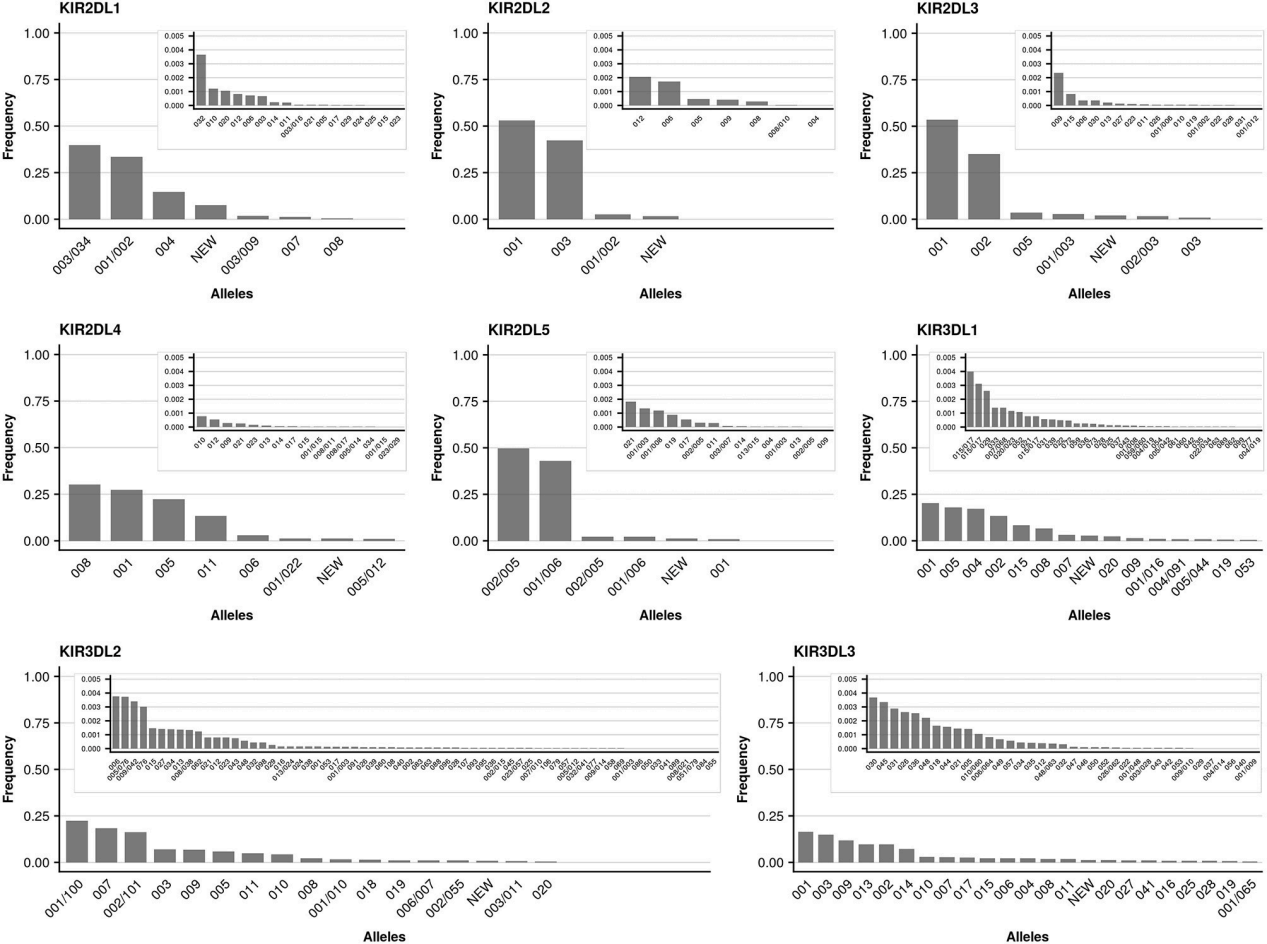
Allele frequencies of inhibiting KIR genes. Allele frequencies (at 3-digit resolution) of inhibiting *KIR* genes based on a dataset of 337,387 samples and KIR-IPD database release 2.7.1. Alleles not resolvable based on the targeted exons are separated by “/.” Due to silent mutations, two 5-digit alleles may separate into distinct 3-digit allele groups with different levels of ambiguities (e.g., KIR2DL3^*^001 and KIR2DL3^*^001/003). Alleles with frequencies below 0.005 are plotted in an inlay.

**Figure 5:**
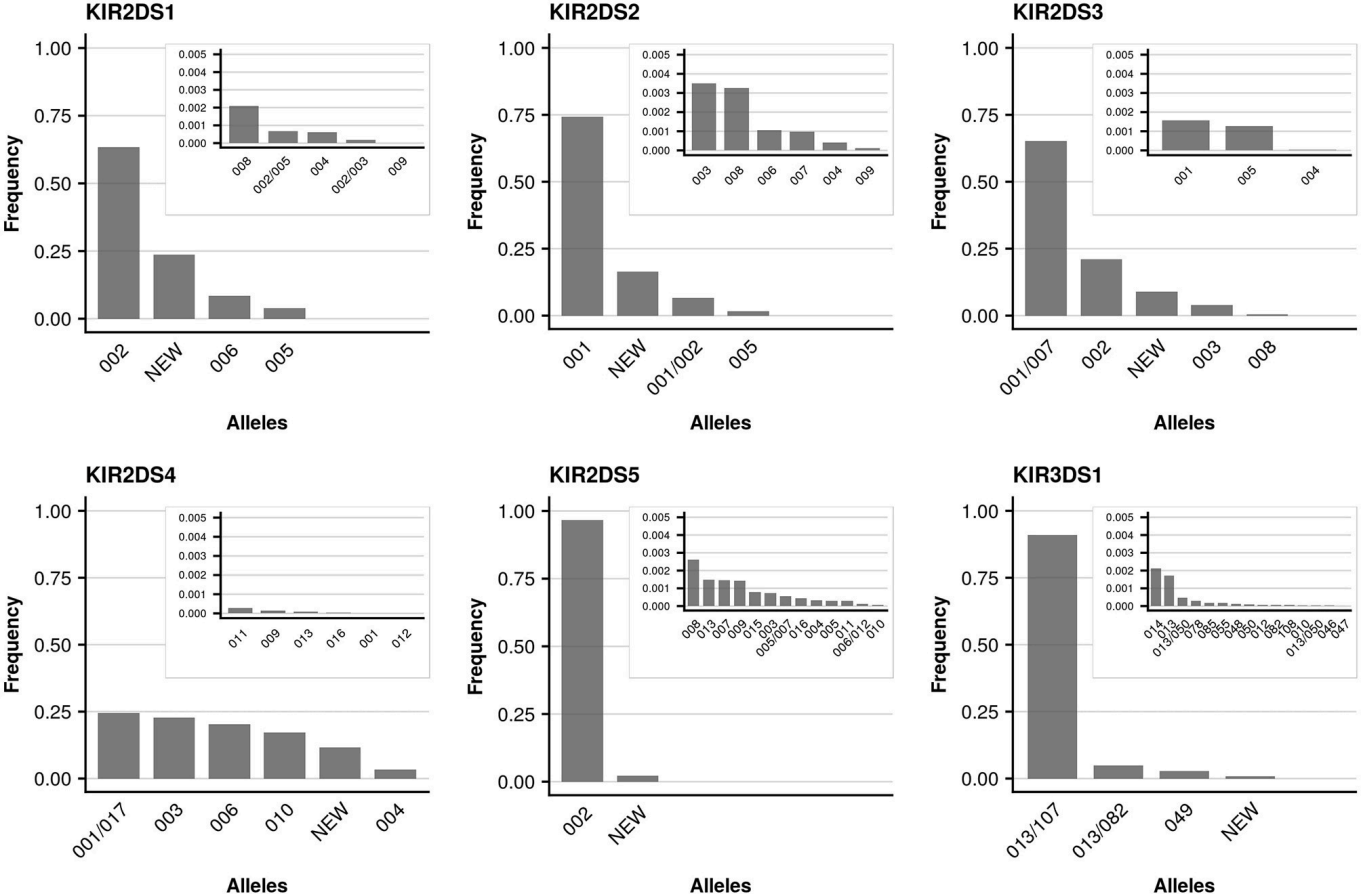
Allele frequencies of activating KIR genes. Allele frequencies (at 3-digit resolution) of activating KIR genes based on a dataset of 337,387 samples and KIR-IPD database release 2.7.1. Alleles not resolvable based on the targeted exons are separated by “/.” Due to silent mutations, two 5-digit alleles may separate into distinct 3-digit allele groups with different levels of ambiguities (e.g., KIR2DS2^*^001 and KIR2DS2^*^001/002). Alleles with frequencies below 0.005 are plotted in an inlay.

We further took advantage of the unprecedented cohort size with respect to *KIR* allele-level typing and determined allele frequencies for all detected *KIR* genes. The individual genes differed markedly with regard to the number of alleles present in the data set, as well as their relative allele frequencies. As reported previously (49), limited allelic diversity was found in the activating *KIR* genes (*KIR2DS1, KIR2DS2, KIR2DS3, KIR2DS4, KIR2DS5, KIR3DS1*) with currently (KIR-IPD 2.7.1) around a dozen (7 to 22) alleles per gene known at the allotype level (Figure 4). In addition to the limited overall diversity, the allele distribution in activating *KIR* genes was heavily skewed: The single most abundant allele of the activating *KIR* genes (with the exception of *KIR2DS4*) accounted for at least 60% (*KIR2DS1*) and up to 97% (*KIR2DS5*) of the sample set. For *KIR2DS4* five common alleles were found at largely similar frequencies. In contrast, the inhibiting *KIR* genes generally displayed more diversity (Figure 5). In particular, for the genes with three Ig-domains (*KIR3DL1, KIR3DL2, KIR3DL3*) many different alleles were identified at rather evenly distributed frequencies. Among genes with two Ig domains, *KIR2DL1* and *KIR2DL4* demonstrated rather broad allele frequency distributions, while *KIR2DL2, KIR2DL3, KIR2DL5* were each dominated by two common alleles having a combined frequency of ~90%.

**Figure 6:**
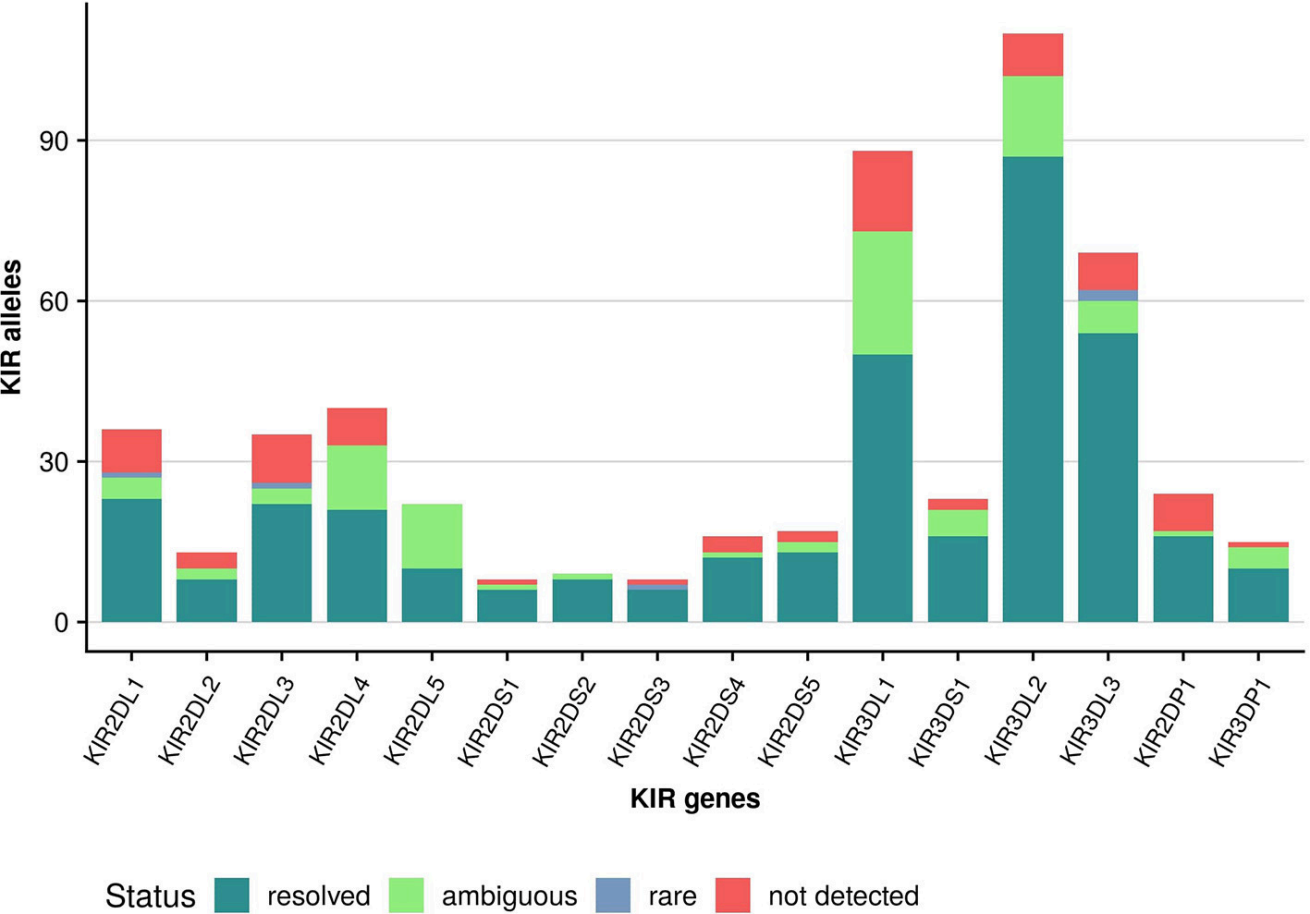
Coverage of IPD-KIR allotypes. KIR alleles (IPD-KIR 2.7.1) were classified at the allotype level (first three digits): unambiguously detected alleles (resolved); alleles detected at a frequency of below 0.00001 (rare); alleles detected as part of an allele group (ambiguous); not detected alleles (not detected).

Owing to the large cohort size we can report allele frequencies for over two thirds (69%) of the alleles described in IPD-KIR 2.7.1 at the protein (allotype) level (Figure 6). For another 16% we can only report the frequency of allele groups which cannot be distinguished by our approach as the differentiating polymorphism lies outside the targeted regions (i.e., in exon 1 or exon 6). However, despite the large cohort size, 15% of the alleles described in IPD-KIR 2.7.1 were not detected. This likely indicates population-specific alleles not represented in our predominantly European cohort, particularly for alleles first identified in samples of African or Asian origin. On the other hand, this may be due to sequencing errors in the original submission. For example, the allele KIR2DS5^*^001 was not detected in our cohort. It differs from KIR2DS5^*^002 (97% frequency) in three positions in exon 4 which are conserved across all other *KIR2DS5* alleles. Likewise, the undetected KIR2DS1^*^001 allele differs from KIR2DS1^*^003 (63% frequency) in one position in exon 3, again conserved across all other *KIR2DS1* alleles.

In conclusion, this analysis provides a comprehensive overview of the alleles and their frequencies to be expected in a European sample set.

### Detection of Novel Alleles

**Figure 7:**
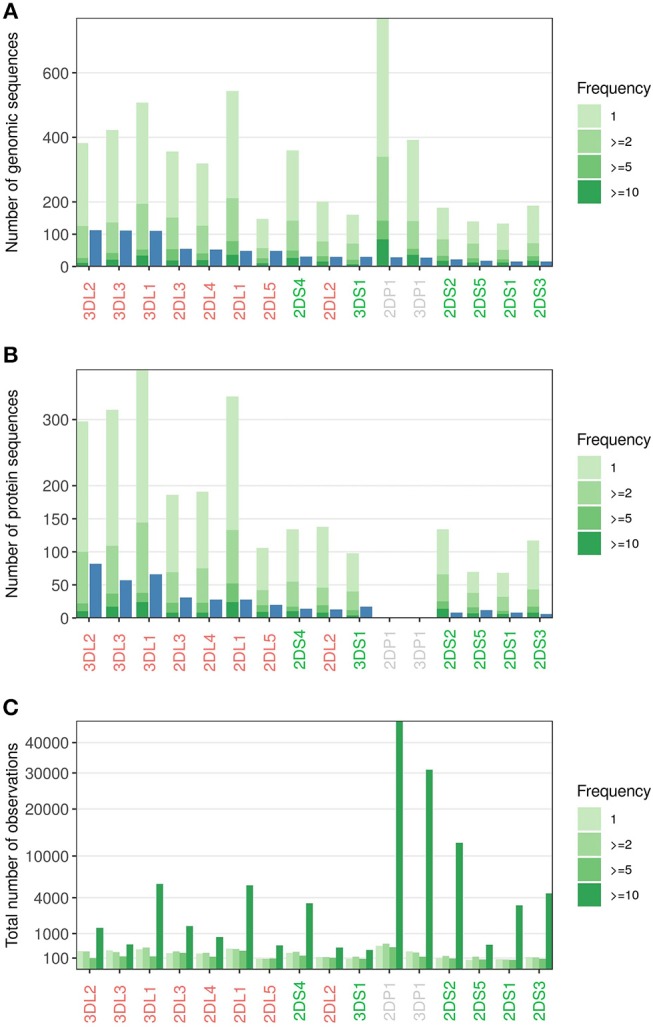
Novel alleles observed. (A) Number of alleles reported in IPD-KIR Release 2.6 (blue) vs. the distinct additional sequences identified in a cohort of 185,170 samples (green). The color code indicates the number of independent observations per novel sequence. (B) As in A, but only alleles/sequences giving rise to distinct protein sequences were counted. (C) Number of samples where any of the novel sequences of A was observed; grouped by the frequencies of the novel sequences. KIR genes are sorted by the number of reported alleles.

To estimate the diversity and frequencies of novel allele variants, we analyzed in more detail a subset of 185,170 samples that were acquired using the IPD-KIR Database Release 2.6 (44) as reference. Considering only sequences which could be assigned to a specific *KIR* gene with high confidence, we identified 5,203 distinct sequences with variations from reported alleles (Figure 7A, Supplementary File 1). We have begun independent validation of these allele sequences using full-gene characterization, for submission to the IPD-KIR Database (ENA accession numbers of the first 376 full-length sequences, Supplementary File 2). However, given the large number of samples yet to process, detailed results from this effort will be reported in a future study. More than 2,000 of the novel sequences were identified in at least two individuals, minimizing the risk of spurious calls. About half of the novel sequences identified encode for novel protein sequences (Figure 7B). Even when considering only the sequences identified in at least five individuals, the number of novel sequences (427) approaches the number of currently named alleles on the protein level (481, IPD-KIR Database Release 2.7) (44). Overall, the number of novel sequences identified for each *KIR* gene reflects the diversity pattern currently reported in the IPD-KIR Database with the inhibiting *KIR* genes (red, Figures 7A,B) displaying a higher variability than the activating *KIR* genes (green, Figures 7A,B). However, in particular for the activating genes, the genetic variability seems to be far greater than currently reported: Considering only sequences observed at least twice, we identified (averaged over all activating *KIR* genes) more than four times as many different protein coding sequences as currently reported. The diversity of *KIR2DL1* and *KIR2DL2* seems underreported as well: While we detected, for example, only 10 of the 13 *KIR2DL2* sequences known at the protein level, we observed 46 distinct so far unreported *KIR2DL2* sequences in at least two independent samples.

All *KIR* genes have in common that the few novel sequences observed more than ten times collectively comprise more samples than the high number of singletons (Figure 7C). The 11 most abundant novel sequences each occur at population frequencies of above 1%. This group included two alleles of the non-expressed genes *KIR2DP1* and *KIR3DP1* with frequencies exceeding 10%, presumably reflecting their limited biological relevance and the concurrently reduced research focus.

In addition to the sequence variations reported above, we frequently encountered samples with novel combinations of known exonic sequences. In other cases, one or several exonic sequences could not be detected. This may partly reflect technical limitations like variations in the primer binding region impeding amplification or insertions/deletions interfering with recognition. However, this may also reflect novel alleles, fusion genes or other genomic reorganizations. Given the quantitative algorithm applied, those special cases were readily detected and flagged. However, the exact description of those sequences requires full gene sequencing and exceeds the capabilities of the described high-throughput workflow.

Taken together, this study confirms that the most abundant alleles are adequately reflected in the database with exception of the pseudogenes harboring undescribed alleles in more than 1/5 individuals. Further, the data sheds light on the high diversity of the *KIR* genes that has only been touched at the surface so far. After integration of the herein described alleles, the IPD-KIR Database should better reflect at least the diversity found in the European population.

## Discussion

The regulation of NK cell activity by the *KIR* gene family is orchestrated by a complex interplay of haplotypes including variation in gene and allele content, NK cell activation (licensing) or inactivation and interaction with HLA molecules, or other unknown ligands. A diverse set of methods has been applied over the last years to unveil the complex genetic diversity (50). Several methods were reported for assaying gene content at the absence/presence level (51–53) or quantitatively (54–56). Others have focused on the allelic characterization of specific *KIR* genes by either sequencing, PCR-SSOP or PCR-SSP (57–60). The comprehensive analysis of all *KIR* genes at allelic resolution until recently required the combination of several assays (61). In a joined study, several groups combined their expertise for the in-depth characterization of a cohort of 512 samples (49). However, only the application of NGS enabled the comprehensive characterization of the complete *KIR* family of genes at allelic level in one assay (29). In contrast to our approach which is based on PCR for target selection, the study by Norman et al. (31)relies on capture technology. Therefore, it is not restricted to selected exons but rather able to provide sequence information covering complete genes including all exons and introns. Despite its holistic nature, the capture assay is applicable to the analysis of large cohorts enabling studies with hundreds of samples, thereby presumably superseding most if not all of the previously published methods. Our approach, however, delivers yet another level of high-throughput capability. We routinely genotype more than 6,000 samples for the complete *KIR* gene family and six *HLA* genes on a single HiSeq run. At the same time, our *KIR* assay is extremely cost-efficient with the main cost drivers being five PCR reactions and about 40,000 HiSeq reads (equaling currently about $1.5 in sequencing costs) per sample. Despite this cost-optimized setup, which presumably is less than even a simple PCR-SSP assay for absence/presence *KIR* genotyping, we obtain allotype-level resolution, including copy number estimations.

The high-throughput data generation process is supported by a highly automated data analysis pipeline. Extensive optimization and validation ensure high-resolution *KIR* genotyping with high accuracy. The software is conservatively tuned, reverting to lower resolution or presence calls whenever a quality parameter indicates an increased risk of spurious results. The main challenge lies in the accurate copy number estimation of the sequence features, in particular for features present in more than two copies. While one and two copies can be estimated with good accuracy, the variance in copy number estimation increases with larger copy numbers often triggering a fallback-to-presence call. In the dataset of 337,387 samples studied here for determining *KIR* allele frequencies, 8% of the results at the individual *KIR* gene level were reported as present. Since these presence calls presumably are not completely random, they entail a potential distortion in the allele frequencies reported here. We are continuously working on reducing the rate of low-resolution genotyping results by analyzing the specific reasons and fine tuning and improving the neXtype software accordingly. However, trained analysts may inspect the warnings and all underlying data, identify inaccuracies and, based on the comprehensive picture, improve the genotyping resolution. Therefore, for clinical samples or research studies of lower numbers (up to several thousands) the workflow and software provide the opportunity for a close-to-complete (>99%) characterization at the allelic level at an extremely low error rate as demonstrated by the accuracy study. Currently, however, this implies that so far unnamed alleles are reported. Analyzing a subset of 185,170 samples, we identified 5,203 distinct novel alleles. This indicates that our current knowledge is only a faint reflection of the true *KIR* diversity. Full-length characterization of even the more frequent of these novel alleles will pose a major challenge. However, after developing whole *KIR* gene amplification assays and updating the appropriate tools for reference sequencing (46–48), we are currently in the process of submitting the first few hundred sequences to the IPD-KIR Database.

The primary motivation for this study was the potential impact of *KIR*-genotype informed donor selection on HSCT outcome. NK cells are thought to mediate potent anti-leukemic effects in the context of allogeneic stem cell transplantation, so called graft-vs.-leukemia reactions (62). When stem cells are transplanted from a healthy donor to a diseased patient, activity of natural killer cells partly depends on the donor compound gene content for activating and inhibitory *KIR* in conjunction with the KIR ligands determined largely by the *HLA* genotype of the patient. Both, the *KIR* B haplotype and the presence of the *KIR2DS1* gene in the donor *KIR* repertoire were associated with a reduced risk of relapse and better relapse-free survival of patients with AML after allogeneic stem cell transplantation (21, 22). However, gene content analysis falls short of depicting the granular levels of interaction introduced by the large variety of *KIR* alleles (63). Both, binding affinity, and protein expression on the cell surface vary between different *KIR* allotypes. For example, disruption of the amino-acid sequence motif WSAPS in *KIR3DL1* by substitution of leucine for serine at position 86 blocks the correct folding of the Ig-like domains and results in retention of the protein in the endoplasmic reticulum (64). The *KIR3DL1* alleles ^*^004, ^*^019, ^*^021, ^*^036, ^*^037, ^*^039, ^*^056, and ^*^072, with ^*^004 being one of the three most common *KIR3DL1* alleles (Figure 4), share this feature and thus are usually not expressed at measurable levels on the cell surface. Variation in expression and density of *KIR3DL1* has been shown to impact on AIDS progression in HIV-positive patients and on antibody-dependent cellular cytotoxicity against neuroblastoma cells (13, 54, 55). Most importantly, preliminary studies indicate that the strength of KIR allele-HLA ligand interaction may impact stem cell transplantation outcome (65). Donor selection incorporating *KIR* allele information may thus positively affect outcome (66). Two large studies addressing the benefits of *KIR*-(allele)-repertoire based donor selection are in progress (NCT02450708 & NCT01288222).

Unfortunately, fully *HLA*-matched donors are still not available for all patients in need for a stem cell transplant. However, as a result of the registration of millions of volunteers by the donor centers worldwide, many potential donors are available for the more abundant genotypes: e.g., at least four *HLA*-compatible 10/10 matched donors are available for the majority of patients with European ancestry. Therefore, a substantial proportion of patients could benefit from considering additional selection criteria. Since the effects on transplantation outcome are driven by the interaction of the KIR repertoire of donor cells with many cognate ligands on recipient hematopoietic cells encoded in the *HLA* class I genes, *KIR* genotyping of the patients is not absolutely required simplifying translation into clinical practice. Therefore, donors may be selected based on KIR/ligand information once high-resolution *KIR* genotyping data is available for a sufficient number of potential stem cell donors. As of August 2018 we have generated *KIR* genotyping data for 3.2 million potential donors, including *KIR* allele-level data for 1.8 million donors. Therefore, for 20% of the globally registered donors (with typing profile of at least *HLA-A, -B, -C, -DRB1*) *KIR* data is already available. Allele-level *KIR* data is currently available for 10% of these donors. Extrapolating the current recruitment rates we project to reach 5 million allele-level *KIR* typed donors by 2021. We hope that these efforts will contribute to translating experimental and clinical research efforts involving different aspects of the *KIR* family genes into clinical practice to further improve outcome of unrelated stem cell transplantation. In addition, availability of such a cost-efficient high-throughput workflow may prove beneficial for the further exploration of *KIR* genes and alleles for population and disease association studies.

## Data Availability Statement

The raw data supporting the conclusions of this manuscript will be made available by the authors, without undue reservation, to any qualified researcher.

## Author Contributions

KL developed and tested the primer sets. IW, DS, JPr, GS, BS, JH, CG, JSa, JPi, and VL conceived the neXtype algorithms. DS, JPr, JH, CG, and JSa implemented neXtype. BS and IW adapted the IPD-KIR allele data for neXtype. BH and NG evaluated the neXtype results. WM, RD, JAH, and PN evaluated the PING results for precision and accuracy estimations. GS, DS, and IW analyzed data and prepared figures and tables. VL, JSc, and GS wrote the manuscript. AS, GS, JSa, JPi, and VL supervised the work. All authors contributed to manuscript revision, read and approved the submitted version.

### Conflict of Interest Statement

IW, GS, BS, NG, KL, BH, AS, and VL are members of the DKMS Life Science Lab which offers commercial *HLA* and *KIR* genotyping services. The remaining authors declare that the research was conducted in the absence of any commercial or financial relationships that could be construed as a potential conflict of interest.

## Supplementary Material

Supplementary File 1 Novel Sequences observed. 185,170 samples were analyzed for sequences deviating from the alleles contained in KIR-IPD 2.6.1. An ID was assigned to each variant. For SNPs within exons of expressed proteins, codon number, reference/variant codon sequences and corresponding amino acids are listed. For SNPs within intron regions, the 10 bases up- and downstream of the SNPs are reported. Variants with several SNPs are described in consecutive lines if the SNPs are not within the reported sequence. Variant bases are color coded in red.

Supplementary File 2 ENA accession numbers of full-length KIR sequences. In case of sequences identified and characterized in several samples, only one submission is referenced.

## Abbreviations

ECN: Exon sequence Copy Number
EAG: Exon Allele Group
GCN: Gene Copy Number
EAGC: EAG Combination
*KIR*: Killer-cell Immunoglobulin-like Receptor.
